# Decisions, Decisions: Observations of Resource Allocation Under Consumer-Directed Care

**DOI:** 10.3390/healthcare13050516

**Published:** 2025-02-27

**Authors:** Danelle Kenny, Kim-Huong Nguyen, Zachary Breig, Lana Friesen, Tracy Comans

**Affiliations:** 1 Centre for Health Services Research, University of Queensland, Herston 4006, Australia; 2 Brain and Mind Center, University of Sydney, Camperdown 2050, Australia; 3 Global Brain Health Institute, Trinity College Dublin, D02 X9W9 Dublin, Ireland; 4 School of Economics, University of Queensland, St Lucia 4072, Australia; 5 National Ageing Research Institute, Parkville 3052, Australia

**Keywords:** consumer-directed care, resource allocation, community-based services, aging in place

## Abstract

**Introduction**: Resource trade-offs are a universal feature of decision-making in healthcare. Public funding for home care is an example of a complex resource allocation decision, requiring balance between the needs of the individual and the capacity of the welfare system to meet those needs across the population. Under consumer-directed care policies, responsibility for resource allocation decisions rests with the care recipient, but there is no existing measure of allocative efficiency resulting from these consumer-led decisions. Our research considers resource allocation decisions by home care package recipients under consumer-directed care and evaluates the consistency of consumer-directed resource allocation with medically identified recommendations. **Method**: Retrospective cohort analysis of twenty home care package resource allocations made by home care package recipients in South-East Queensland, compared to associated medically identified needs and discussions with a potential service provider. **Findings**: Resource allocation decisions in the Australian home care setting are complex, multi-faceted, and made in highly uncertain environments. There are significant differences between services and equipment recommendations made by assessment teams, service providers, and home care recipient choices. **Discussion**: Awareness of the decision-making process and resulting decisions provides a foundation for future research to simplify decision-making for home care package recipients without sacrificing autonomy, leading to improved resource allocation efficiency and home care program outcomes. **Conclusion**: Consumer-directed care is a globally popular policy position to allocate public funds related to health care needs, despite uncertainty around the impact of this policy on program outcomes. Our analysis suggests further understanding of factors influencing resource allocation decisions is needed to define appropriate supports for effective decision-making in home care resource allocation, and further research effort is required to determine efficient resource allocation to inform policy changes, irrespective of the decision-maker.

## 1. Introduction

### 1.1. Significance

We live in a world of resource scarcity, forcing trade-offs and difficult decisions in every aspect of life. Healthcare is far from exempt. Many developed countries have identified substantial social and economic benefits to ageing in place, but there is scant research into resource allocation efficiency and how this might vary under different policies. From a government expenditure perspective, programs are funded with the assumption that decision-makers and market mechanisms will allocate resources efficiently. In reality, biases, beliefs, preferences, uncertainty, and contextual factors lead to widely disparate resource allocations among different actors, indicating that selecting the decision-maker is itself a crucial policy choice. As an example, our research examines resource allocation decisions made by Australian home care package recipients under consumer-directed care and evaluates the consistency of their decisions with medical assessment of need.

#### 1.1.1. Australian Aged Care System

The Australian aged care system is a network of services and programs designed to support people to maintain a healthy lifestyle as they age [1]. Entry into the aged care system is facilitated through My Aged Care, a multi-platform gateway designed to provide a single point of entry to aged care services, though referral pathways are varied. There are two main settings for aged care, community-based (homecare) and facility-based (residential aged care), each having different types of programs and levels of support. Eligibility for the type of package and level of support a person can receive is determined through an Aged Care Assessment Team (ACAT) assessment. An ACAT assessment is triggered by the person applying for aged care services, or a person involved in their care, such as a friend, a family member, or a health professional (general practitioner, allied health, or hospital staff). ACAT assessments determine eligibility for packages and an appropriate setting of care and recommend the level of funding a recipient should receive. ACAT assessments are purely based on identified service and equipment needs, they do not allocate service providers, consider service availability in the recipient’s geographical location, or show any sensitivity to wait times between the assessment and receipt of services. Under consumer-directed care, this information is sourced by the package recipient themselves.

#### 1.1.2. Home Care

The home care package program is a community-based program designed to support people with complex needs and circumstances to age-in-place, i.e., independently in their own homes [2]. Home care package recipients in Australia are older citizens (65+) assessed as needing assistance to remain living independently at home. Currently, there are four levels representing thresholds corresponding with the level of support needed as determined by the ACAT assessment. As levels increase, so does the funding available for services and equipment.

The home care program has been subject to several iterations, directed by different policy foci and with different approaches to resource allocation [3]. In 2012, it was determined that home care packages would be distributed under consumer-directed care arrangements, with all home care packages distributed this way from 2015, with funds directly allocated to the person receiving the package, a deviation from the previous model where fund distribution was controlled by service providers. Each new version of the program has steered toward increasing choice and flexibility for older people needing care.

#### 1.1.3. Consumer-Directed Care

Consumer-directed care is a policy position that aims to empower ‘consumers’ (end-users) of health services by endowing them with the final choice in how package resources are allocated [4]. Its adoption is increasing across many developed countries as it purportedly increases both flexibility and autonomy for home-based care recipients [5]. Mechanisms for implementing the policy vary across countries and jurisdictions, with cash-for-care being the predominant scheme [3]. The effect of this is the creation of a quasi-market mechanism as opposed to government-controlled service provision. There has been limited evaluation of consumer-directed care with respect to outcomes of welfare programs or the effect of consumer-directed care on resource allocation decisions in any jurisdiction [6].

In essence, consumer-directed care transfers decision-making responsibility from service providers and governments to package recipients. The intention of this transfer is to align the needs and preferences of the recipient with resource allocation, resulting in more efficient resource distribution. In practice, the efficiency of resource distribution under any home care policy is unknown, and recipient’s short-term choices do not exclusively align with long term health goals.

#### 1.1.4. Resource Allocation Decision-Making

The choices presented to home care package recipients under consumer-directed care are resource allocation decisions, even though they are not necessarily perceived or approached as such. The decision environment is deceptively complex, requiring home care package recipients to select appropriate equipment and services to facilitate ageing in place under conditions of uncertainty, with large volumes of sometimes conflicting information and the pressure to make multiple decisions quickly. Resource allocation decisions are difficult, with many competing factors increasing risk of adverse outcomes and little information to indicate which particular mix of services will result in positive program outcomes. Under such conditions, cognitive processes seek to optimize effort and mitigate time constraints, real or perceived, leading to snap decisions and limited attention to all decision-making factors [7]. Whilst this approach to decision-making is efficient, it can often lead to judgement errors, in this case resulting in sub-optimal resource allocation, i.e., selecting services that do not delay or prevent transfer to residential aged care.

### 1.2. Purpose

The purpose of our observations is to describe resource allocation decisions made by home care package recipients under consumer-directed care, and compare these against assessed needs, as identified by the ACAT assessment. Additionally, we consider the conversations between service providers and home care package recipients, and the potential influence these discussions have on final choices.

## 2. Materials and Methods

### 2.1. Study Design and Setting

The study design follows a retrospective cohort framework, applying observational research methodology. Home care packages are assigned to individual recipients, and decisions around allocating these funds occur during meetings or discussions with service providers (onboarding meetings). From September to December 2021, we observed twenty onboarding meetings between an onboarding officer representing a South-East Queensland sub-branch of a large Australian home care provider, newly allocated home care package recipients, and their nominated support person/s. Onboarding meetings were conducted in the home care package recipient’s home.

We combined transcripts of the observed resource allocation decisions with additional data including ACAT assessments, government correspondence, photographs of the environment, transcripts from follow-up interviews, and recipient care-plans. Ten recipients also provided further information immediately following resource allocation, which helped to further elucidate thinking behind the decisions.

### 2.2. Sampling and Consent

Convenience sampling was used whereby any newly approved home care recipient approaching the service provider was asked to participate in the study. The service provider approached each recipient and referred interested people to the research team. The primary fieldwork researcher then contacted the person by phone to explain the project and obtain verbal consent. A written copy of the participant information sheet and consent form was then e-mailed and/or posted, depending on the preference of the package recipient. A second copy was taken by the fieldwork researcher to the onboarding meeting, and written consent obtained from all participants (including home care package recipient and any support persons). Participants were encouraged to ask questions and were free to withdraw from the study at any time prior to transcription of the meeting recordings.

### 2.3. Data Sources

Principal data are sourced from transcripts of twenty onboarding meetings between a single client liaison officer and their interactions with individual home care package recipients. Each transcript was allocated a randomly generated numerical identifier (ID) to anonymise the data while preserving capacity to analyse resource allocation at the individual level. Most meetings were attended by a family member, either on the phone or in person. One or two members of the research team attended each meeting, obtained consent, and recorded the discussion.

Discussions were lengthy and the service provider stipulated researcher involvement could not interfere with the discussion, so any questions posited by researchers to home care package recipients were to occur after their discussion. As such, the application of real-time think-aloud techniques were not possible and involvement of the researcher in the discussion varied. While all sessions were recorded, in half of the sessions (10) the researcher purely observed the recipient allocating resources, and in the other half, researchers posed additional questions immediately following the onboarding discussion. Questions were delivered in a semi-structured interview conducted by the attending researcher. These questions related to individual approaches to decisions in a retrospective think-aloud style, asking decision-makers to give voice to their ‘thinking’ when selecting services to clarify how decisions were made. The decision to interview half using the think-aloud protocol was multi-factorial, including keeping transcript length manageable, reducing fatigue for the home care package recipient, providing the opportunity to identify potential bias introduced by the researchers’ questions (e.g., did think-aloud lead recipients to consider their choice in a different way) and to manage time for the service provider representative.

The primary researcher conducted follow up phone-calls with the package decision-maker 6–8 weeks after the interview, recording and assessing any changes to the decisions made during the observed session, adding a robust, longitudinal element to the data [8]. Primary source supporting documentation was added to the dataset, including photographs of the home environment and filing systems, flowcharts made by home care package recipients, acceptance letters, official government correspondence, price lists, and ACAT assessments. Transcription of interviews and follow-up conversations was completed by the primary researcher with the assistance of otter.ai© software. De-identified transcripts were then uploaded and stored on a secure data storage platform.

### 2.4. Calculation of Resource Allocation

For each resource allocation observed, services and equipment; 1—recommended by ACAT, 2—discussed by the service provider and home care package recipient during the interview, and 3—chosen by package recipients, were recorded. Using the budgeting tool available to the service provider representative and the package level offered to the recipient, each resource allocation variation was costed and used for comparisons:

- Budget Percentage: Costs for each service category expressed as a percentage of the allowed budget at the offered level.
- Proportional Allocation: Percentage of total costs recommended for each service category, ignoring budget level.
- Unmet Need: Cost of full coverage according to medically assessed need subtracted from level approved and level offered.

## 3. Results

### 3.1. Descriptive Data

Characteristics of participants are described in Supplementary File S1. Participants entering the home care package program ranged from 68–98 years, with a median age of 82. All participants were living in south-east Queensland at the time of their discussion, though there was a mix of urban and rural locations within the provider’s service area. There was variation in socio-economic status as measured by the income tested fee calculation, a means-tested payment determined by the government to be the amount recipients can afford to contribute toward their care. Most package recipients were female (80%), slightly higher than the national average of 66% (1). Most packages were approved at level 2 (50%), followed by level 3 (35%), and level 4. No participants were approved at level 1, though seven package recipients were offered interim packages at a lower level.

### 3.2. Complexity of the Health Care System

All participants had previous experience with the aged care system and had received some service or supports at home under various programs, including Community Home Support Program (a precursor to home care with a small amount of services funded by government), transition care (an intense rehabilitation program provided to people returning home from hospital), or community services from the home and community care programs (a federally funded health program for frail, aged Australians and their carers). Previous experience of the aged care system and pathway into aged care were varied, including a review or revision of existing assessments, referred by family, self-referred, referred on discharge from hospital, or referred by visiting allied health professionals attending under other programs.

### 3.3. Decision Support

Most participants attended the session with a nominated decision-support person (90%), even if they retained the capacity to make their own decisions. Half of the home care recipients made the final decision about their resource allocation. Recipients’ children proxied in 35% of cases and recipients’ spouses in 15%. Several participants had advanced symptoms of dementia, and a proxy decision-maker was appointed through enduring power of attorney. Our analysis concentrates on the decision outcome and person making the decision, irrespective of whether the decision-maker was the home care package recipient or a proxy.

### 3.4. Decision-Making Environment

During the onboarding discussion, home care package recipients must choose the services and equipment required to support and maintain their independence. They need to allocate funding across five domains: complexity and vulnerability, medical, physical, psychological, and social. Concurrent choices include which service provider will administer their package (participants were encouraged to compare providers) [2], whether additional fees are payable, including the income tested fee, and how these costs compare to existing expenses, and trade-offs between services and equipment [3].

Consumer-directed care creates a competitive market in which service-providers compete for recipients. This competition induces a proliferation of marketing materials with heterogeneous designs that make comparing providers more difficult. Additionally, providers have different fee structures and conditions of service that complicate comparisons, such as charging per hour or by 15 min blocks, so while the hourly rate listed is less, a recipient may be charged for an hour when the service was only received for 15 min.

Government agencies and advocacy programs aiming to inform recipients of their approvals and information about the package add to recipients’ confusion. There are numerous ambiguities, including the importance of the information in the correspondence and the person to whom the correspondence is addressed. Abundant information is supplied to recipients, but this does not correlate with accessibility and is often too general to be specific or relevant to the individual, thereby increasing the volume of information without increasing understanding. Consequently, correspondence from government agencies like Centrelink are often mixed with marketing materials and stored haphazardly, accumulating on coffee tables or being swept into a drawer, resulting in difficulty locating important details, such as payable fees, when decisions need to be made.

Further environmental pressures are created by time constraints. While the service provider was careful to remind recipients that they could take time to think about the decision and didn’t need to decide on the day, recipients felt they needed to ‘lock in’ their package; assuming if they did not decide quickly, they would lose the package altogether. The duration of the resource allocation discussions varied from 35 min to 2.5 h.

### 3.5. Resource Allocation Calculation

Resource allocation, percentage of budget, proportional allocation of services within budget, and unmet need calculations for each package recipient are included as Supplementary File S1. We present summary and aggregate findings below, beginning with the average proportion of allocated funds to types of services, followed by the proportion and value of the approved budget allocated by ACAT assessment, service providers, and home care package recipients, and concluding with details of identified unmet needs associated with interim care packages.

When using pre-defined service categories from the service provider’s budgeting tool, the average proportional allocation across all package recipients shows differences between ACAT recommendations, discussions with the service provider, and final choices (Table 1).

In general, Community and Support Services, which include personal care (showering), cleaning, gardening, shopping, meal preparation, and social support are most recommended by all, though the proportion recommended by assessed needs is lower than the amount chosen (60.54% vs. 65%). Nursing services are recommended by ACAT assessors more often than service providers (19.84% vs. 14%) but only included in 11% of final home care package service selections. Allied Health Services are frequently recommended by ACAT assessment and service providers (13.95% and 15%, respectively) but chosen by home care package recipients rarely (3%).

Considering budget percentage calculations, both ACAT assessment and discussions with the service provider generate recommendations that cost more than the approved subsidy, whereas recipients are more likely to only accept services up to the level of funding provided, avoiding any out-of-pocket costs (Table 2).

ACAT recommendations are derived from a lengthy, medically oriented assessment tool. The recommendations reflect the health status of the applicant at the time the assessment is made and suggest services and equipment necessary to support the applicant to remain living independently at home. The number of services and intensity of need determine the level of home care package offered (Level 1–4), with higher levels indicating higher or more complex needs and attracting a higher subsidy. On occasion, the identified needs are significantly higher than what can be provided (Table 3). In this situation, package recipients are forced into a trade-off, needing to decide if they are willing to incur out-of-pocket costs or choose between their needs. Of the observed cohort, ten recipients had ACAT recommendations exceeding the approved level of funding, these deficits are greater for people offered interim packages (packages at a lower level than approved).

The service provider recommends services and equipment commonly provided to home care package recipients, beginning with the services the recipient is already receiving and then inviting the recipient to add services or equipment as the budget allows. In the absence of specific requests from the recipient, the service provider is observed to prompt the recipient from a pre-defined list. This list appears to be specific to the provider budget tool and not directly linked to the recipient’s ACAT assessment. There is a complicated interplay between entitlements at different levels and across different programs that is absent from ACAT assessments.

The observed final resource allocation closely conforms to service provider recommendations delivered during the onboarding discussion (Table 4). Recipients already receiving services from the provider tended toward accepting the home care package, retaining the service provider, and maintaining the existing services being provided. Any additional services were likely to have been suggested by the service provider during the onboarding discussion. Many services and equipment items recommended in the ACAT assessment are not discussed by the service provider or chosen by home care package recipients. Of the 172 ACAT recommendations made to package recipients, less than half were discussed in interviews and only 44 were eventually chosen.

At times, a package at the approved level was not available, and an interim package at a lower level was offered (Table 5). This generates unmet needs, where package recipients rely on alternative funding or incur out-of-pocket costs to meet their needs. There were 7 recipients out of 20 (35%) offered interim packages. Of these 7 recipients, 6 were receiving pensions and no one chose to fund the gap out-of-pocket.

In summary, our analysis confirms vulnerable home care package recipients are tasked with complex resource allocation decisions under conditions of uncertainty and with limited formal decision support. On average, the majority of home care package funding is allocated to community and support services, with high variability of nursing and allied health services being recommended compared to being selected. On average, recommendations by both ACAT assessment and service providers exceeded the funded portion of home care packages, highlighting resource scarcity and the presence of trade-offs in resource allocation decisions for home care packages under consumer-directed care. Home care package recipients were more likely to overlook ACAT recommendations in favour of service provider recommendations and generally chose fewer services than recommended by ACAT assessment, especially medically related services such as nursing and allied health. Recipients were unlikely to make decisions resulting in out-of-pocket costs and offered interim packages at lower levels resulted in high levels of unmet need, at an average of $1463.77 per month.

## 4. Discussion

The purpose of describing our observations was to map resource allocation decisions made by home care package recipients under consumer-directed care and compare these decisions against assessed needs, as identified by the ACAT assessment. At the decision-point, it is likely that home care package recipients are influenced by the presentation of information during multi-purpose discussions, where package recipients are concurrently deciding whether to accept a package, whether to accept terms of service from that provider, and what services or equipment will allow them to age well in their own homes. As such, we examined the content of discussions between service providers and home care package recipients that framed the decision environment for the home care package recipient as the decision-maker for resource allocation under consumer-directed care.

### 4.1. Decision Environment

Human decision-making is often sub-conscious and inherently complex (7). Aspects of the environment surrounding the decision-maker can often help or hinder effective decision-making. Many of the observed environmental factors in the onboarding discussions can be interpreted to complicate the decision-making process, such as time, uncertainty, and the number of concurrent decisions.

There is a building evidence base that time pressure affects decision-making, with shorter durations tending toward riskier choices, potentially as a consequence of truncated information processing capacity [9]. We can understand this behaviour as the tendency to make decisions using mental shortcuts (heuristics) that produce a potentially different decision outcome than a carefully weighted decision resulting from additional time to consider all contributing factors. With an average duration of 45 min to make multiple decisions, there is, arguably, insufficient time for home care package recipients to truly consider their long-term needs and the appropriate service-mix to meet those needs.

Uncertainty is one of the largest contributors to decision complexity [10]. Defined as the absence of information, uncertainty obfuscates optimal decisions. The circumstances leading to a home care package initiation are varied and highly uncertain. Many people receiving the package do not know what their future health needs will be or what period of time exists between their current health state and a potentially worse one. Additional uncertainty bleeds in from service availability, waiting periods, and budgetary constraints. This additional stress reduces cognitive capacity to make reasoned, informed decisions.

The decision-making environment is further complicated by the number of choices recipients are required to make in the short time frame. Defined as a deterioration of quality in decision-making, decision fatigue describes a negative correlation between the number of decisions a person makes and the energy remaining to make subsequent decisions [11]. In the home care package setting, recipients are asked to make several concurrent decisions under a cloud of uncertainty and subject to time pressures. Decisions include whether to accept the package, which service provider, what waiting periods, what services, what program, what trade-offs, and what budgetary constraints. Under these conditions, decision-making shortcuts are likely.

### 4.2. Information Volume, Complexity, and Consistency

One of the underlying tenets of healthcare is informed consent, where care cannot be accepted if recipients are not fully aware of all possible risks and benefits. Both legally and ethically, a person is not able to make decisions about their medical care unless they have sufficient information and understanding. This extends to healthcare services provided under government funding. Coupled with the ease of modern technology to generate large volumes of information, home care package recipients receive a lot of information. Unfortunately, the information is inconsistently distributed, sometimes conflicting, and the amount of information is often excessive. The principle of providing complete information has the opposite effect of its intention, resulting in a state of cognitive overload where recipients are unable to process all of the information presented. This cognitive state of being overwhelmed often leads to decision deferral or maintaining the status quo [12].

### 4.3. Resource Allocation

The intention of consumer-directed care is to drive empowered decision-making by recipients (4), who know their needs best; however, our observations suggest a significant dependence on service provider recommendations, which may not align with either the client preferences or care needs. Understanding the effect of resource allocation on program outcomes is critical to program success [13]. How resource allocation varies between decision-makers and the subsequent impact on outcomes associated with these choices are necessary data points to achieve efficiency. This is important for policymakers to consider when designing government funded programs, because the allocation of resources influences program outcomes. Further understanding of optimal resource allocation in home care is required to ensure the policy remains consistent with its objectives.

Additional attention should be directed to the recommendations of the service provider and reasons why their recommendations differ from the ACAT assessment [14]. There is a possibility that the ACAT assessment is outdated and no longer reflects the true needs of the recipient, and a further possibility that service provider representatives are encouraged to recommend a service-mix that optimises the number and level of services that the provider specialises in rather than what is most needed by the recipient. Alternatively, there may be financial constraints that limit the recipient’s ability to implement the full suite of ACAT recommendations, and trade-offs occur to remain within the limits of government funding.

Finally, consideration is given to the ACAT recommended resource allocation. While the ACAT assessment is the existing standard and is understood to identify the services and equipment required to support home care recipients to age in place, there is currently no evaluation of home care packages that identifies optimal resource allocation. In the absence of this evidence, it is not possible to say which service-mix should be encouraged.

### 4.4. Limitations

Developing a deep understanding of decisions requires a qualitative approach that has trade-offs for generalisability. We acknowledge a small sample size drawn from a narrow geographic area; this is appropriate for qualitative research, but a larger sample is needed for reducing the uncertainty of quantitative estimates. We also acknowledge that service provider interactions include only one representative from the service provider. It is possible that other service providers or other staff of the service provider may conduct onboarding protocols differently to those observed. Acknowledging this, we draw no further generalisations to the broader population but recommend further research to investigate any possibility of homogeneity in these observed patterns across different countries and cultures. We submit this research as a contribution to the field to further our understanding of resource allocation under consumer-directed care policy.

## 5. Conclusions

Resource allocation is a global consideration that presents challenging decisions for policymakers, particularly in health care settings. In many countries, consumer-directed care is a popular policy position that allocates resources according to patient needs, given the assumption that the health consumer best understands their needs. In Australia, consumer-directed care was introduced to protect the autonomy and dignity of older Australians receiving home care packages, affording final decisions on resource allocation to the person receiving care. Whether this policy has been advantageous for the home care package program is debated.

Our observations of resource allocation under consumer-directed care show that the combination of services and equipment eventually selected by the final decision-maker varies substantially from medically identified needs forming the basis of funding allocation. This has several implications for resource allocation in home care packages or similar public welfare programs and the policy underpinning the selection of the decision-maker. Principally, awareness and understanding of underlying factors driving decision outcomes in complex decision environments needs to inform the selected policy governing resource allocation, and steps taken to reduced decision complexity should be incorporated into the policy framework. Second, further evaluation of the home care package program in relation to the overarching objective of the program is required to better understand what combinations of services and equipment best meet the program objectives, irrespective of the decision-maker determining resource allocation.

## Figures and Tables

**Table 1:** Average Proportion of Resource Allocation by Category.

RESOURCE CATEGORY	ACAT	SP	FINAL
Comm. & Support Services	60.54%	59%	65%
Nursing Services	19.84%	14%	11%
Allied Health Services	13.95%	15%	3%
Transport Individual Vehicle Costs	3.19%	2%	0%
Transport Group Vehicle Costs	0.00%	0%	0%
Centre Bus Travel	0.05%	0%	0%
Brokered Services	0.00%	0%	13%
Consumables	2.43%	10%	8%
CHECK	100.00%	100.00%	100%

Table notes: ACAT = Aged Care Assessment Team assessment, SP = Service Provider, FINAL = Package Recipient choice, Comm. = Community, Brokered Services = Services delivered to package recipient by external providers through agreement with service provider.

**Table 2:** Average Allocated Budget (Proportion) by Agent.

RESOURCE CATEGORY	ACAT	SP	FINAL
Comm. & Support Services	86%	77%	36%
Nursing Services	34%	19%	8%
Allied Health Services	27%	18%	1%
Transport Individual Vehicle Costs	4%	3%	0%
Transport Group Vehicle Costs	0%	0%	0%
Centre Bus Travel	0%	0%	0%
Brokered Services	0%	0%	13%
Consumables	3%	8%	3%
Fees	0%	22%	18%
Total	111%	126%	69%

Table notes: ACAT = Aged Care Assessment Team assessment, SP = Service Provider, FINAL = Package Recipient choice, Comm. = Community, Brokered Services = Services delivered to package recipient by external providers through agreement with service provider.

**Table 3:** Cost per Month of ACAT Recommendations vs. Level of Funding.

ID	ACAT$	Package Level$	DIFF
171105	$6562.19	$4813.74	−$1748.45
206366	ALT	ALT	$0.00
213762	$3042.00	$3212.30	$170.30
247581	$5113.00	$2847.91	−$2265.09
270339	$2957.00	$4813.74	$1856.74
298570	$1482.50	$1308.83	−$173.67
300631	$3845.85	$2847.91	−$997.94
412122	$1723.50	$2847.91	$1124.41
483101	$2472.50	$1308.53	−$1163.97
483249	$1987.75	$1308.83	−$678.92
493062	$1762.50	$1308.83	−$453.67
503135	$1972.50	$1308.83	−$663.67
544638	$2730.25	$3175.05	$444.80
630860	$859.75	$4317.34	$3457.59
795603	$962.50	$1308.83	$346.33
830024	$1019.25	$1308.83	$289.58
830937	$2401.50	$1459.39	−$942.11
879341	$1621.50	$2847.91	$1226.41
916234	$2359.00	$4813.74	$2454.74
959965	$1421.00	$1308.83	−$112.17

Table notes: ID = Randomly generated client identifier to show individual cases; ACAT$ = Value of services and equipment recommended by ACAT assessment; Package Level$ = Dollar Value provided by government at allocated package level and including dementia subsidy where applicable; DIFF = Difference between identified need and funding allocated; ALT—Recipient accepted package with alternate provider and did not provide budgeting information; Dollar Values per month for package level 1 = $743.99, level 2 = $1308.83, level 3 = $2847.91, level 4 = $4317.34, dementia supplement = +11.5%.

**Table 4:** Comparison of Assessed Need, Discussion, and Choice.

Need vs. Choice	Recommended	Discussed	Chosen
ID			
*TOTAL*	*172*	*77*	*44*
171105	16	6	3
206366	8	3	1
213762	8	3	1
247581	7	4	2
270339	6	4	1
298570	5	2	2
300631	16	3	4
412122	10	3	3
483101	10	5	2
483249	8	3	2
493062	9	3	2
503135	11	9	3
544638	11	4	5
630860	6	4	1
795603	4	3	3
830024	4	4	2
830937	8	5	2
879341	10	4	3
916234	8	2	0
959965	7	3	2

Table Notes: ID = Randomly generated client identifier to show individual cases; Recommended = Explicit service or equipment recommendation made by ACAT assessor; Discussed = Explicit service or equipment suggestion made during discussion with service provider; Chosen = Explicit service or equipment choice made by home care package recipient as part of home care package (from follow up interview).

**Table 5:** Unmet Need per Month from Interim Packages.

ID	Needs	Approved	Offered	Difference	Unmet Need
171105	$6562.19	$4317.34	$1308.83	$3008.51	−$5253.36
298570	$1482.50	$1308.83	$743.99	$564.84	−$738.51
412122	$1723.50	$2847.91	$743.99	$2103.92	−$979.51
483249	$1987.75	$1308.83	$743.99	$564.84	−$1243.76
493062	$1762.50	$1308.83	$743.99	$564.84	−$1018.51
830024	$1019.25	$1308.83	$743.99	$564.84	−$275.26
879341	$1621.50	$2847.91	$743.99	$2103.92	−$877.51
SUM	$16,159.19	$15,248.48	$5772.77	$9475.71	−$10,386.42
AVG	$2308.46	$2178.35	$824.68	$1353.67	−$1483.77

Table Notes: ID = Randomly generated client identifier to show individual cases; Needs = Value of explicit equipment and services identified in ACAT assessment; Approved = Value of funding allowed in package; Offered = Value of interim package offered to home care package recipient while waiting for package at appropriate level to become available; Difference = Gap between value of approved package and value of offered package; Unmet Need = Gap between value of identified needs and value of offered package.

## Data Availability

The data presented in this article are not readily available because they contain potentially identifiable information and are protected under privacy legislation. Deidentified data informing the analysis are provided in Supplementary File S1.

## Supplementary Materials

The following supporting information can be downloaded at: https://www.mdpi.com/article/10.3390/healthcare13050516/s1, File S1: Resource allocation by individual.
