# Supplementary material for: Decisions, Decisions: Observations of Resource Allocation Under Consumer-Directed Care
Source: Healthcare (Basel). 2025 Feb 27;13(5):516. doi: 10.3390/healthcare13050516 (PMC11899287; doi:10.3390/healthcare13050516)
Supplement: Supplementary file 1 [file healthcare-13-00516-s001.zip › healthcare-3444199-supplementary.pdf]

SUPPLEMENTARY FILE S1

SUMMARY TABLE

ID	Age	Sex	Lvl	Lvl	Signed	Decision-m	Family	SES (ITF)	Referred from	Y	ACAT $	Approved $	DIFF
171105	89	F	L4	L2	Y	HCP	Y	Pension	Family	N	$ 6,562.19	$ 4,813.74	-$1,748.45
206366	88	F	L3	L3	ALT	DTR	Y	Pension	Daughter	Y			$0.00
213762	71	F	L3	L3	Y	HCP	Y	Pension	Self	Y	$ 3,042.00	3212.3	$170.30
247581	73	F	L3	L3	N	HCP	Y	ITF (?)	Doctor	Y	$ 5,113.00	$2,847.91	-$2,265.09
270339	79	F	L4	L4	Y	SON	Y	Pension	Hospital	Y	$ 2,957.00	$ 4,813.74	$1,856.74
298570	74	M	L2	L1	N	HCP	N	Pension	Hospital	N	$ 1,482.50	$ 1,308.83	-$173.67
300631	75	F	L3	L3	Y	HCP	Y	Pension	Self	Y	$ 3,845.85	$2,847.91	-$997.94
412122	68	F	L3	L1	Y	HCP	Y	Pension	OT	N	$ 1,723.50	$ 2,847.91	$1,124.41
483101	82	F	L2	L2	Y	HCP	Y	Pension	Nurse	Y	$ 2,472.50	1308.53	-$1,163.97
483249	86	F	L2	L1	Y	HCP	Y	Pension	TCP	N	$ 1,987.75	$ 1,308.83	-$678.92
493062	84	F	L2	L1	ALT	HUS	Y	ITF ($1.43)	Nurse	N	$ 1,762.50	$ 1,308.83	-$453.67
503135	98	M	L2	L2	Y	SON	Y	ITF ($8.20)	OT	Y	$ 1,972.50	$1,308.83	-$663.67
544638	74	F	L3	L3	N	HCP	N	ITF ($13.00)	OT	Y	$ 2,730.25	$3,175.05	$444.80
630860	91	M	L4	L4	ALT	DTR	Y	Pension	Daughter	Y	$ 859.75	$4,317.34	$3,457.59
795603	76	M	L2	L2	Y	WIF	Y	Pension	Wife/Carer	Y	$ 962.50	$1,308.83	$346.33
830024	95	F	L2	L1	Y	DTR	Y	Pension	TCP	N	$ 1,019.25	$ 1,308.83	$289.58
830937	90	F	L2	L2	ALT	SON	Y	Pension	Hospital	Y	$ 2,401.50	$1,459.39	-$942.11
879341	83	F	L3	L1	Y	SON	Y	Pension	Son	N	$ 1,621.50	$ 2,847.91	$1,226.41
916234	74	F	L4	L4	ALT	HUS	Y	ITF ($35.00)	Nurse	Y	$ 2,359.00	$4,813.74	$2,454.74
959965	92	F	L2	L2	Y	HCP	Y	Pension	Nurse	Y	$ 1,421.00	$1,308.83	-$112.17

Explanatory Notes: Age (years); Sex at birth, F=Female, M=Male; Lvl App, Approved funding allocation for home-care package Level 1-4 as stipulated in Aged Care Assessment Team recommendation; Lvl Off, Level of funding offered at time of discussion (aka interim package); Signed – whether the home-care package was accepted, Y – signed with interviewing service provider, ALT – signed with alternate service provider, N-declined package; Decision-maker HCP – Home-care package recipient, DTR – Daughter, SON – Son, WIF – Wife, HUS, Husband; Family Y – family member present for allocation discussion, N – No family support for decision-making; SES (ITF) Socio-Economic Status (Income Tested Fee), Pension – home-care package recipient receives a government pension with no additional income, ITF – Income tested fee payable (amount to be paid daily); Referred from – person or entity that submitted referral to My Aged Care, OT – Occupational Therapist, TCP – Transition Care Program.

Interpreting Resource Alocation: ID - Randomised client identifier; ACAT Recommendation - Service type and priority contained in Aged Care Assessment; Budget Allocation Discussion - Service type recommended by service provider during onboarding interview; Resource Allocation Decision - Choice made by home care package recipient; Service Category - Expense category as defined by service provider budgeting tool; Budget percentage - cumulated costs by service category expressed as a percentage of the total budget of OFFERED package level; Proportional allocation - Shows proportion allocated to service category as a total of services recommended (ignores budget level); Unmet need: For people offered interim packages, shows total cost of medically recommended services (ACAT) and whether interim and/or approved budget meets identified needs.

ID	ACAT Recommendation (priority)	Service Category	Budget Allocation Discussion	Service Category	Resource Allocation Decision	Service Category
171105	HCPL4 (high)	HCP Level	Fees	Fees	HCPL2	HCP Level
	Meals - delivered (high)	Consumables and Equipment	Bathroom renovations	Consumables and Equipment	Social visit 1hr/wk	Comm & Support Services
	Nursing (high)	Nursing Services	Continence aids	Alternate Funding - CAPS (L2 pkg)	Domestic 1.5hr/wk	Comm & Support Services
	Home mods (high)	Comm. & Support Services	Taxi Vouchers	Transport - Individual	Hygiene 45min/wk	Comm & Support Services
	Food prep at home (med)	Allied Health Services	Respite	Comm & Support Services		
	OT (med)	Allied Health Services	Showering	Comm & Support Services		
	Physio (med)	Comm. & Support Services	Physio	Allied Health Services		
	Showering (med)	Comm. & Support Services	OT	Allied Health Services		
	Gardening (med)	Allied Health Services	Equipment (shower chair)	Consumables and Equipment		
	Dietitian (med)	Allied Health Services				
	Social work (med)	Comm. & Support Services				
	Domestic (med)	Comm. & Support Services				
	Dementia support (med)	Comm. & Support Services				
	Social support -group (low)	Comm. & Support Services				
	Respite (low)	Comm. & Support Services				
	Equipment (low)	Consumables and Equipment				
**Budget percentage (of HCPL2)**	**$1,308.83**	**Budget %**	**$**	**Budget %**	**$**	**Budget %**
Comm. & Support Services	$ 3,917.44	268.40%	$ 1,951.00	133.69%	$ 1,051.25	72.03%
Nursing Services	$ 948.75	65.00%	$ -	0.00%	$ -	0.00%
Allied Health Services	$ 1,408.00	96.50%	$ 1,164.00	79.76%	$ -	0.00%
Transport Individual Vehicle Costs	$ 58.00	3.80%	$ 56.00	3.84%	$ 56.00	3.84%
Transport Group Vehicle Costs	$ -	0.00%	$ -	0.00%	$ -	0.00%
Centre Bus Travel	$ 54.00	3.70%	$ -	0.00%	$ -	0.00%
Brokered-in Services from other service providers	$ -	0.00%	$ -	0.00%	$ -	0.00%
Consumables	$ 176.00	12.10%	$ 176.00	12.06%	$ -	0.00%
Fees	$ -	0.00%	$ 307.58	21.08%	$ 307.58	21.08%
TOTAL	$ 6,562.19	-349.50%	$ 3,654.58	-150.42%	$ 1,414.83	3.05%
**Proportional Allocation**						
Comm. & Support Services	59.70%		58%		95%	
Nursing Services	14.46%		0%		0%	
Allied Health Services	21.46%		35%		0%	
Transport Individual Vehicle Costs	0.88%		2%		5%	
Transport Group Vehicle Costs	0.00%		0%		0%	
Centre Bus Travel	0.82%		0%		0%	
Brokered-in Services from other service providers	0.00%		0%		0%	
Consumables	2.68%		5%		0%	
CHECK	*100.00%*		*100%*		*100%*	
**Unmet Needs**	**$**	**L2**	**L4**			
Funding Allocation		$ 1,459.39	$ 4,813.74			
Comm. & Support Services	$ 3,917.44	$ 1,051.25				
Nursing Services	$ 948.75	$ -				
Allied Health Services	$ 1,408.00	$ -				
Transport Individual Vehicle Costs	$ 58.00	$ 56.00				
Transport Group Vehicle Costs	$ -	$ -				
Centre Bus Travel	$ 54.00	$ -				
Brokered-in Services from other service providers	$ -	$ -				
Consumables	$ 176.00	$ -				
TOTAL	$ 6,562.19	$ 1,107.25				
Budget deficit/surplus		-$ 5,102.80	-$ 1,748.45			

ID	ACAT Recommendation (priority)	Service Category	Budget Allocation Discussion	Service Category	Resource Allocation Decision	Service Category
213762	HCP L3	HCP Level	Fees	Fees	Domestic Support x2 hours	Community and Support Services
	Accompanied Shopping (high)	Comm. & Support Services	Shopping	Social Support	Showers x30min	Community and Support Services
	Occupational Therapy (High)	Allied Health Services	Other providers	-	Exercise	Allied Health Services
	Direct Transport (Medium)	Comm. & Support Services	Equipment (O2 concentrator)	Consumables	Oxygen	Consumables
	Taxi Vouchers (Medium)	Transport	Continence	Consumables	Incontinence Pads	Consumables
	General house cleaning (Medium)	Comm. & Support Services	Transport	Transport - Individual		
	Assistance with self care (Low)	Comm. & Support Services	Gardening	Community and Support Services		
	CAS (Low)	Nursing Services	Palliative care options	Community and Support Services		
	General repairs (low)	Comm. & Support Services	Domestic Support	Community and Support Services		
			Medication	Nursing Services		
			Higher package	Funding		

**Budget percentage (of HCPL3)**	**$ 3,212.30**	**Budget %**	**$ 3,212.30**	**Budget %**	**$ 3,212.30**	**Budget %**
Comm. & Support Services	$ 1,533.25	48%	$ 1,269.25	40%	$ 674.00	21%
Nursing Services	$ 119.25	4%	$ 1,068.00	33%	$ -	0%
Allied Health Services	$ 122.00	4%	$ -	0%	$ 488.00	15%
Transport Individual Vehicle Costs	$ 112.50	4%	$ 140.00	4%	$ -	0%
Transport Group Vehicle Costs	$ -	0%	$ -		$ -	0%
Centre Bus Travel	$ -	0%	$ -	0%	$ -	0%
Brokered Services	$ -	0%	$ -	0%	$ -	0%
Consumables	$ 1,155.00	36%	$ 1,155.00	36%	$ 1,155.00	36%
Fees	$ -	0%	$ 669.26	21%	$ 669.26	21%
**TOTAL**	**$ 3,042.00**	**95%**	**$ 4,301.51**	**134%**	**$ 2,986.26**	**93%**

**Proportional Allocation**			
Comm. & Support Services	50%	35%	29%
Nursing Services	4%	29%	0%
Allied Health Services	4%	0%	21%
Transport Individual Vehicle Costs	4%	4%	0%
Transport Group Vehicle Costs	0%	0%	0%
Centre Bus Travel	0%	0%	0%
Brokered Services	0%	0%	0%
Consumables	38%	32%	50%
CHECK	*100%*	*100%*	*100%*

ID	ACAT Recommendation (priority)	Service Category	Budget Allocation Discussion	Service Category	Resource Allocation Decision	Service Category
247581	Shopping (high)	Comm. & Support Services	Existing Services		Declined package	
	Respite (med)	Comm. & Support Services	2x daily insulin injection	Nursing Services	remains on CHSP	
	Medication (med)	Nursing Services	Fortnightly shopping	Comm. & Support Services	daily nursing - medication	Nursing Services
	Transport (med)	Comm. & Support Services	Fees	Comm. & Support Services	fortnightly shopping	Comm. & Support Services
	Nursing (med)	Nursing Services	Cleaning	Comm. & Support Services		
	Domestic (low)	Comm. & Support Services	Bathroom Mods			
	Home Modification (low)	Allied Health Services	Shower Rail			

**Budget percentage (HCPL3)**	**$2,847.91**	**Budget %**	**$**	**Budget %**	**$**	**Budget %**
Comm. & Support Services	$ 1,148.25	40.32%	$ 714.75	25.10%	$ 257.00	9.02%
Nursing Services	$ 3,702.75	130.02%	$ 2,846.25	99.94%	$ 2,846.25	99.94%
Allied Health Services	$ 122.00	4.28%	$ -		$ -	0.00%
Transport Individual Vehicle Costs	$ 140.00	4.92%	$ -		$ -	0.00%
Transport Group Vehicle Costs	$ -		$ -		$ -	0.00%
Centre Bus Travel	$ -		$ -		$ -	0.00%
Brokered Services	$ -		$ -		$ -	0.00%
Consumables	$ -		$ -		$ -	0.00%
Fees	$ -		$ 669.25	23.50%	$ -	0.00%
TOTAL	$ 5,113.00	179.54%	$ 4,230.25	148.54%	$ 3,103.25	108.97%

**Proportional Allocation**			
Comm. & Support Services	22.46%	20%	8%
Nursing Services	72.42%	80%	92%
Allied Health Services	2.39%	0%	0%
Transport Individual Vehicle Costs	2.74%	0%	0%
Transport Group Vehicle Costs	0.00%	0%	0%
Centre Bus Travel	0.00%	0%	0%
Brokered Services	0.00%	0%	0%
Consumables	0.00%	0%	0%
CHECK	*100.00%*	*100%*	*100%*

**Unmet Needs**	**$**	**L3**	**CHSP**	
Funding Allocation				NOTE: Equipment - Bathroom mods and Shower Rail approx $5500
Comm. & Support Services				
Nursing Services				
Allied Health Services				
Transport Individual Vehicle Costs				
Transport Group Vehicle Costs				
Centre Bus Travel				
Brokered Services				
Consumables				
TOTAL	$ 5,113.00	$ -		
Budget deficit/surplus		-$ 5,113.00	-$ 5,113.00	

ID	ACAT Recommendation (priority)	Service Category	Budget Allocation Discussion	Service Category	Resource Allocation Decision	Service Category
270339	Personal care (high)	Comm. & Support Services	Existing services (showering, home mods)	Comm. & Support Services	Hygiene 3x30min/wk	Comm. & Support Services
	Respite (med)	Comm. & Support Services	Help with walking	Comm. & Support Services	Social support 1.5hr/fn	Comm. & Support Services
	OT (med)	Allied Health Services	Nursing	Nursing Services		
	Meals (med)	Comm. & Support Services	Social outings	Comm. & Support Services		
	Home mods (med)	Equipment	Domestic support	Comm. & Support Services		
	Nursing (low)	Nursing	Meals	Consumables		
			Physio	Allied Health Services		
			Continence	Consumables		
			Gardening	Comm. & Support Services		

**Budget percentage (of HCPL4 + dem supp)**	**$ 4,813.74**	**Budget %**	**$**	**Budget %**	**$**	**Budget %**
Comm. & Support Services	$ 2,715.75	56.42%	$ 1,252.00	26.01%	$ 561.50	11.66%
Nursing Services	$ 119.25	2.48%	$ 119.25	2.48%	$ -	0.00%
Allied Health Services	$ 122.00	2.53%	$ 488.00	10.14%	$ -	0.00%
Transport Individual Vehicle Costs	$ -		$ -	0.00%	$ -	0.00%
Transport Group Vehicle Costs	$ -		$ -	0.00%	$ -	0.00%
Centre Bus Travel	$ -		$ -	0.00%	$ -	0.00%
Brokered Services	$ -		$ -	0.00%	$ -	0.00%
Consumables	$ -		$ 286.00	5.94%	$ -	0.00%
Fees	$ -		$ 1,014.57	21.08%	$ 1,014.57	21.08%
TOTAL	$ 2,957.00	61.43%	$ 3,159.82	65.64%	$ 1,576.07	32.74%

**Proportional Allocation**			
Comm. & Support Services	91.84%	58%	100%
Nursing Services	4.03%	6%	0%
Allied Health Services	4.13%	23%	0%
Transport Individual Vehicle Costs	0.00%	0%	0%
Transport Group Vehicle Costs	0.00%	0%	0%
Centre Bus Travel	0.00%	0%	0%
Brokered Services	0.00%	0%	0%
Consumables	0.00%	13%	0%
CHECK	*100.00%*	*100.00%*	*100.00%*

Equipment - Home Modifications (Bathroom Renovations) = $30,000

ID	ACAT Recommendation (priority)	Service Category	Budget Allocation Discussion	Service Category	Resource Allocation Decision	Service Category
298570	Nursing (high)	Nursing Services	Existing services (showering & cle	Comm. & Support Services	Declined package	
	Domestic (med)	Comm. & Support Services	Nursing	Nursing Services	CHSP	
	Podiatry (med)	Allied Health Services	Domestic Assistance	Comm. & Support Services	showers 3 x 30min weekly	Comm. & Support Services
	Home Mods (low)	Comm. & Support Services	Shopping	Comm. & Support Services	domestic cleaning 1hr/fn	Comm. & Support Services
	CAS (low)	Nursing Services	Budget Restrictions	Fees		

**Budget percentage (of HCPL1)**	**$743.99**	**Budget %**	**$**	**Budget %**	**$**	**Budget %**	
Comm. & Support Services	$ 361.25	48.56%	$ 1,019.25	137%	$ 674.00	90.59%	
Nursing Services	$ 975.75	131.15%	$ 856.50	115%	$ -	0.00%	
Allied Health Services	$ 145.50	19.56%	$ -	0%	$ -	0.00%	
Transport Individual Vehicle Costs	$ -	0.00%	$ -	0%	$ -	0.00%	
Transport Group Vehicle Costs	$ -	0.00%	$ -	0%	$ -	0.00%	
Centre Bus Travel	$ -	0.00%	$ -	0%	$ -	0.00%	
Brokered Services	$ -	0.00%	$ -	0%	$ 1,073.33	144.27%	Wound Care alternate provider
Consumables	$ -	0.00%	$ -	0%	$ -	0.00%	
Fees	$ -	0.00%	$ 307.58	41%	$ -	0.00%	
TOTAL	$ 1,482.50	199.26%	$ 2,183.33	293.46%	$ 1,747.33	234.86%	

**Proportional Allocation**			
Comm. & Support Services	24.37%	54%	39%
Nursing Services	65.82%	46%	0%
Allied Health Services	9.81%	0%	0%
Transport Individual Vehicle Costs	0.00%	0%	0%
Transport Group Vehicle Costs	0.00%	0%	0%
Centre Bus Travel	0.00%	0%	0%
Brokered Services	0.00%	0%	61%
Consumables	0.00%	0%	0%
CHECK	*100.00%*	*100.00%*	*100.00%*

**Unmet Needs**	**$**	**CHSP**	**L2**
Funding Allocation			$ 1,308.83
Comm. & Support Services			
Nursing Services			
Allied Health Services			
Transport Individual Vehicle Costs			
Transport Group Vehicle Costs			
Centre Bus Travel			
Brokered Services			
Consumables			
TOTAL	$ 1,482.50	$ -	
Budget deficit/surplus		-$ 1,482.50	-$ 173.67

ID	ACAT Recommendation (priority)	Service Category	Budget Allocation Discussion	Service Category	Resource Allocation Decision	Service Category
300613	Nursing (high)	Nursing Services	Grief counselling	Allied Health Services	Domestic 90 min/week	Brokered
	Home Maintenance (high)	Comm. & Support Services	Cleaning	Comm. & Support Services	OT Assessment	Allied Health Services
	Meals (High)	Consumables	Doorbell	Equipment	Podiatrist	Allied Health Services
	Occupational Therapy (high)	Allied Health Services	Fees	Fees	Taxi Vouchers	Consumables
	Personal Care (high)	Comm. & Support Services	Transport vouchers	Consumables		
	Domestic Assistance (med)	Comm. & Support Services				
	Transport (med)	Consumables				
	Social Support (med)	Comm. & Support Services				
	Home Mods (low)	Equipment				
	Podiatry (low)	Allied Health Services				
	Physio (low)	Allied Health Services				
	Speech Pathology (low)	Allied Health Services				
	Respite (low)	Comm. & Support Services				
	Residential Aged Care (low)	Comm. & Support Services				
	Hydrotherapy (low)	Allied Health Services				
	Social Work (low)	Allied Health Services				

**Budget percentage (of HCPL3)**	**$2,847.91**	**Budget %**	**$**	**Budget %**	**$**	**Budget %**
Comm. & Support Services	$ 1,445.25	50.75%	$ 168.75	5.93%	$ -	0.00%
Nursing Services	$ 856.50	30.07%	$ -	0.00%	$ -	0.00%
Allied Health Services	$ 1,321.75	46.41%	$ 197.00	6.92%	$ 194.75	6.84%
Transport Individual Vehicle Costs	$ -	0.00%	$ -	0.00%	$ -	0.00%
Transport Group Vehicle Costs	$ -	0.00%	$ -	0.00%	$ -	0.00%
Centre Bus Travel	$ 6.75	0.24%	$ -	0.00%	$ -	0.00%
Brokered Services	$ -	0.00%	$ -	0.00%	$ 371.25	13.04%
Consumables	$ 215.60	7.57%	$ 110.00	3.86%	$ 110.00	3.86%
Fees	$ -	0.00%	$ 669.25	23.50%	$ 669.25	23.50%
TOTAL	$ 3,845.85	135.04%	$ 1,145.00	40.20%	$ 1,345.25	47.24%

**Proportional Allocation (HCPL3)**		
Comm. & Support Services	37.58%	35%	0%
Nursing Services	22.27%	0%	0%
Allied Health Services	34.37%	41%	29%
Transport Individual Vehicle Costs	0.00%	0%	0%
Transport Group Vehicle Costs	0.00%	0%	0%
Centre Bus Travel	0.18%	0%	0%
Brokered Services	0.00%	0%	55%
Consumables	5.61%	23%	16%
CHECK	100.00%	100.00%	100.00%

**Unmet Needs**	**$**	**L2**	**L4**
Funding Allocation			
Comm. & Support Services			
Nursing Services			
Allied Health Services			
Transport Individual Vehicle Costs			
Transport Group Vehicle Costs			
Centre Bus Travel			
Brokered Services			
Consumables			
TOTAL	$ 3,845.85	$ -	
Budget deficit/surplus		-$ 3,845.85	-$ 3,845.85

ID	ACAT Recommendation (priority)	Service Category	Budget Allocation Discussion	Service Category	Resource Allocation Decision	Service Category
412122	Occupational Therapy (med)		Existing Services		HCPL3 accepted	
	Physio (med)		Physiotherapy		Equipment	
	Personal Care (low)		Provider comparisons		Physiotherapy – brokered	
	Podiatry (low)		Meals		Podiatry – brokered	
	Home Modification (low)				Domestic Cleaning	
	Home Repairs (low)					
	Domestic Cleaning (low)					
	Social Support (low)					
	Transport (low)					
	Meals (low)					

**Budget percentage (of HCPL1)**	**$**	**743.99**	**Budget %**	**$**		**Budget %**	**$**		**Budget %**
Comm. & Support Services	$	1,195.75	160.72%	$	489.75	65.83%	$	200.75	26.98%
Nursing Services	$	-	0.00%	$	-	0.00%	$	-	0.00%
Allied Health Services	$	485.75	65.29%	$	291.00	39.11%	$	-	0.00%
Transport Individual Vehicle Costs	$	42.00	5.65%	$	-	0.00%	$	-	0.00%
Transport Group Vehicle Costs	$	-	0.00%	$	-	0.00%	$	-	0.00%
Centre Bus Travel	$	-	0.00%	$	-	0.00%	$	-	0.00%
Brokered Services	$	-	0.00%	$	-	0.00%	$	270.88	36.41%
Consumables	$	-	0.00%	$	-	0.00%	$	-	0.00%
Fees	$	-	0.00%	$	174.84	23.50%	$	174.84	23.50%
TOTAL	$	1,723.50	231.66%	$	955.59	128.44%	$	646.47	86.89%

**Proportional Allocation**			
Comm. & Support Services	69.38%	63%	43%
Nursing Services	0.00%	0%	0%
Allied Health Services	28.18%	37%	0%
Transport Individual Vehicle Costs	2.44%	0%	0%
Transport Group Vehicle Costs	0.00%	0%	0%
Centre Bus Travel	0.00%	0%	0%
Brokered Services	0.00%	0%	57%
Consumables	0.00%	0%	0%
CHECK	100.00%	100%	100%

**Unmet Needs**	**$**	**L3**	**L1**	**Equipment**
Funding Allocation		$ 2,847.91	$ 743.99	4WW
Comm. & Support Services	$ 1,195.75			Grabrail
Nursing Services	$ -			Glasses
Allied Health Services	$ 485.75			Shower Chair
Transport Individual Vehicle Costs	$ 42.00			
Transport Group Vehicle Costs				
Centre Bus Travel				
Brokered Services				
Consumables				
TOTAL	$ 1,723.50	$ -		
Budget deficit/surplus		$ 1,124.41	-$ 979.51	

Equipment purchase $1100

ID	ACAT Recommendation (priority)	Service Category	Budget Allocation Discussion	Service Category	Resource Allocation Decision	Service Category
483101	Accompanied Shopping (high)	. & Support Services	Shopping	m & Support Services	Domestic Cleaning	m & Support Services
	Vision Services (med)	llied Health Services	Showering	m & Support Services	Shopping	m & Support Services
	In home food prep (med)	. & Support Services	Transport	m & Support Services	Kettle	Equipment
	Shopping Delivery (med)	. & Support Services	Eye Exam (sunglasses)	Allied Health		
	Occupational Therapy (med)	llied Health Services	Social Support	m & Support Services		
	Assisted self-care (med)	Nursing Services	Domestic Cleaning	m & Support Services		
	Direct Driver Transport (low)	. & Support Services	Incontinence	Nursing		
	Domestic Assistance (low)	. & Support Services	Trade-offs (CPAP v services)	Equipment		
	Home Modifications (low)	. & Support Services	Meals	Consumables		
	Gardening (low)	. & Support Services	Yard Maintenance	m & Support Services		
			Mould Remover	m & Support Services		
			Spring Cleaning	m & Support Services		
			Ramp	Equipment		

**Budget percentage (of HCPL2)**		**1308.53**	**Budget %**	**$**		**Budget %**	**$**		**Budget %**
Comm. & Support Services	$	1,268.50	96.94%	$	1,906.63	145.71%	$	457.75	34.98%
Nursing Services	$	948.75	72.51%	$	119.25	9.11%	$	-	
Allied Health Services	$	171.25	13.09%	$	49.25	3.76%	$	-	
Transport Individual Vehicle Costs	$	84.00	6.42%	$	84.00	6.42%	$	-	
Transport Group Vehicle Costs	$	-	0.00%	$	-	0.00%	$	-	
Centre Bus Travel	$	-	0.00%	$	-	0.00%	$	-	
Brokered Services	$	-	0.00%	$	-	0.00%	$	-	
Consumables	$	-	0.00%	$	330.00	25.22%	$	-	
Fees	$	-	0.00%	$	307.58	23.51%	$	307.58	23.51%
TOTAL	$	2,472.50	188.95%	$	2,796.71	213.73%	$	765.33	58.49%

**Proportional Allocation**			
Comm. & Support Services	51.30%	77%	100%
Nursing Services	38.37%	5%	0%
Allied Health Services	6.93%	2%	0%
Transport Individual Vehicle Costs	3.40%	3%	0%
Transport Group Vehicle Costs	0.00%	0%	0%
Centre Bus Travel	0.00%	0%	0%
Brokered Services	0.00%	0%	0%
Consumables	0.00%	13%	0%
CHECK	100.00%	100.00%	100.00%

**Unmet Needs**			
Funding Allocation			
Comm. & Support Services			
Nursing Services			
Allied Health Services			
Transport Individual Vehicle Costs			
Transport Group Vehicle Costs			
Centre Bus Travel			
Brokered Services			
Consumables			
TOTAL		$ -	
Budget deficit/surplus		$ -	$ -

ID	ACAT Recommendation (priority)	Service Category	Budget Allocation Discussion	Service Category	Resource Allocation Decision	Service Category
483249	Personal Care (low)	Comm. & Support Services	Cleaning	Comm. & Support Services	Domestic Cleaning	Comm. & Support Services
	Direct Transport (low)	Comm. & Support Services	Shopping	Comm. & Support Services	Shopping	Comm. & Support Services
	Shopping (low)	Comm. & Support Services	CAS	Nursing Services		
	Continence Advisory Service (low)	Nursing Services	Nutrition	Allied Health Services		
	Nursing (low)	Nursing Services				
	Physiotherapy (low)	Allied Health Services				
	Home Modifications (low)	Comm. & Support Services				
	Domestic Assistance (low)	Comm. & Support Services				

Budget percentage (of HCPL1)	$743.99	Budget %	$	Budget %	$	Budget %
Comm. & Support Services	$ 1,011.00	135.89%	$ 401.50	53.97%	$ 401.50	
Nursing Services	$ 404.75	54.40%	$ 119.25	16.03%	$ -	0.00%
Allied Health Services	$ 488.00	65.59%	$ 122.00	16.40%	$ -	0.00%
Transport Individual Vehicle Costs	$ 84.00	11.29%	$ -	0.00%	$ -	0.00%
Transport Group Vehicle Costs	$ -	0.00%	$ -	0.00%	$ -	0.00%
Centre Bus Travel	$ -	0.00%	$ -	0.00%	$ -	0.00%
Brokered Services	$ -	0.00%	$ -	0.00%	$ -	0.00%
Consumables	$ -	0.00%	$ -	0.00%	$ -	0.00%
Fees	$ -	0.00%	$ 174.84	23.50%	$ -	0.00%
TOTAL	$ 1,987.75	267.17%	$ 817.59	109.89%	$ 401.50	0.00%

Proportional Allocation					
Comm. & Support Services	50.86%		62%		100%
Nursing Services	20.36%		19%		0%
Allied Health Services	24.55%		19%		0%
Transport Individual Vehicle Costs	4.23%		0%		0%
Transport Group Vehicle Costs	0.00%		0%		0%
Centre Bus Travel	0.00%		0%		0%
Brokered Services	0.00%		0%		0%
Consumables	0.00%		0%		0%
CHECK	100.00%	0.00%	100.00%	0.00%	100.00%

Unmet Needs	$	L1	L2
Funding Allocation		$ 743.99	$ 1,308.83
Comm. & Support Services	$ 1,011.00		
Nursing Services	$ 404.75		
Allied Health Services	$ 488.00		
Transport Individual Vehicle Costs	$ 84.00		
Transport Group Vehicle Costs			
Centre Bus Travel			
Brokered Services			
Consumables			
TOTAL	$ 1,987.75		
Budget deficit/surplus		-$ 1,243.76	-$ 678.92

ID	ACAT Recommendation (priority)	Service Category	Budget Allocation Discussion	Service Category	Resource Allocation Decision	Service Category
493062	HCP L2 (med)	N/A	Interim Package L1	N/A	Cooking	Comm. & Support Services
	Respite (med)	Comm. & Support Services	Cooking	Comm. & Support Services	Shopping	Comm. & Support Services
	Home Modification (med)	Comm. & Support Services	Shopping	Comm. & Support Services	Domestic Cleaning	Comm. & Support Services
	Domestic linen (mod)	Comm. & Support Services	Cleaning	Comm. & Support Services		
	Social Support (med)	Comm. & Support Services	Showers	Comm. & Support Services		
	Meals (med)	Comm. & Support Services	Dementia Support	Nursing Services		
	Transport (med)	Transport	Transport	Transport Individual		
	House Cleaning (med)	Comm. & Support Services	Taxi Vouchers	Consumables		
	Residential Aged Care (low)	Comm. & Support Services				
	Shopping (low)	Comm. & Support Services				

**Budget percentage (of HCPL1)**	**$743.99**	**Budget %**	**$**	**Budget %**	**$**	**Budget %**
Comm. & Support Services	$ 1,678.50	225.61%	$ 1,741.75	234.11%	$ 867.00	116.53%
Nursing Services	$ -	0.00%	$ 119.25	16.03%	$ -	0.00%
Allied Health Services	$ -	0.00%	$ -	0.00%	$ -	0.00%
Transport Individual Vehicle Costs	$ 84.00	11.29%	$ 84.00	11.29%	$ -	0.00%
Transport Group Vehicle Costs	$ -	0.00%	$ -	0.00%	$ -	0.00%
Centre Bus Travel	$ -	0.00%	$ -	0.00%	$ -	0.00%
Brokered Services	$ -	0.00%	$ -	0.00%	$ -	0.00%
Consumables	$ -	0.00%	$ -	0.00%	$ -	0.00%
Fees	$ -	0.00%	$ 174.84	23.50%	$ 174.84	23.50%
TOTAL	$ 1,762.50	236.90%	$ 2,119.84	284.93%	$ 1,041.84	140.03%

**Proportional Allocation**			
Comm. & Support Services	95.23%	90%	100%
Nursing Services	0.00%	6%	0%
Allied Health Services	0.00%	0%	0%
Transport Individual Vehicle Costs	4.77%	4%	0%
Transport Group Vehicle Costs	0.00%	0%	0%
Centre Bus Travel	0.00%	0%	0%
Brokered Services	0.00%	0%	0%
Consumables	0.00%	0%	0%
CHECK	100.00%	100.00%	100.00%

**Unmet Needs**	**$**	**L1**	**L2**
Funding Allocation		$ 743.99	$ 1,308.83
Comm. & Support Services	$ 1,678.50		
Nursing Services	$ -		
Allied Health Services	$ -		
Transport Individual Vehicle Costs	$ 84.00		
Transport Group Vehicle Costs			
Centre Bus Travel			
Brokered Services			
Consumables			
TOTAL	$ 1,762.50	$ -	
Budget deficit/surplus		-$ 1,018.51	-$ 453.67

ID	ACAT Recommendation (priority)	Service Category	Budget Allocation Discussion	Service Category	Resource Allocation Decision	Service Category
503135	HCP L2	N/A	Existing Services		Fortnightly housework	Comm. & Support Services
	Domestic Cleaning (high)		Fortnightly housework		Fortnightly shopping	Comm. & Support Services
	Equipment (high)	Equipment	Fortnightly shopping		Meals	Comm. & Support Services
	Accompanied Shopping (med)		Meals		Powerdrive Wheelchair	Equipment
	Physiotherapy (med)		Occupational Therapy			
	Home Modification (med)		Fees			
	Occupational Therapy (med)		Waiting Periods			
	Gardening (low)		Medication side-effects			
	Personal Care (low)		Hip Arthritis (pain)			
	Nursing (low)		Medication management			
	Transport (low)		Transport			
	Social Support (low)		Wheelchair			
			Home Modifications			
			Showers			
			Allied Health			
			Gardening			
			Spring cleaning			
			TENS Machine			
			Physiotherapy			

**Budget percentage (of HCPL2)**	**$1,308.83**	**Budget %**	**$**	**Budget %**	**$**	**Budget %**
Comm. & Support Services	$ 1,356.25	103.62%	$ 1,155.50	88.28%	$ 401.50	30.68%
Nursing Services	$ 119.25	9.11%	$ -	0.00%	$ -	0.00%
Allied Health Services	$ 413.00	31.55%	$ 854.00	65.25%	$ -	0.00%
Transport Individual Vehicle Costs	$ 84.00	6.42%	$ 84.00	6.42%	$ -	0.00%
Transport Group Vehicle Costs	$ -	0.00%	$ -	0.00%	$ -	0.00%
Centre Bus Travel	$ -	0.00%	$ -	0.00%	$ -	0.00%
Brokered Services	$ -	0.00%	$ -	0.00%	$ -	0.00%
Consumables	$ -	0.00%	$ 176.00	13.45%	$ 176.00	13.45%
Fees	$ -	0.00%	$ 307.58	23.50%	$ 307.58	23.50%
TOTAL	$ 1,972.50	150.71%	$ 2,577.08	196.90%	$ 885.08	67.62%

**Proportional Allocation**						
Comm. & Support Services	68.76%		51%		70%	
Nursing Services	6.05%		0%		0%	
Allied Health Services	20.94%		38%		0%	
Transport Individual Vehicle Costs	4.26%		4%		0%	
Transport Group Vehicle Costs	0.00%		0%		0%	
Centre Bus Travel	0.00%		0%		0%	
Brokered Services	0.00%		0%		0%	
Consumables	0.00%		8%		30%	
CHECK	100.00%	0.00%	100.00%	0.00%	100.00%	

**Unmet Needs**	$			**NOTE: Unspent funds go to Equipment (powerdirve chair)**
Funding Allocation				
Comm. & Support Services				
Nursing Services				
Allied Health Services				
Transport Individual Vehicle Costs				
Transport Group Vehicle Costs				
Centre Bus Travel				
Brokered Services				
Consumables				
TOTAL		$ -		
Budget deficit/surplus		$ -	$ -	

ID	ACAT Recommendation (priority)	Service Category	Budget Allocation Discussion	Service Category	Resource Allocation Decision	Service Category
544638	HCPL3 (med)	NA	Existing Services (6days)	Comm. & Support Services	Continuing with CHSP	
	Social Support (high)	Comm. & Support Services	Medication management	Allied Heatlh Services	Showers	Comm. & Support Services
	Nursing (high)	Nursing Services	Showers	Comm. & Support Services	Shopping	Comm. & Support Services
	Dietitian (high)	Allied Health Services	Shopping	Comm. & Support Services	Meals	Consumables
	Occupational Therapist (high)	Allied Health Services	Social Group	Comm. & Support Services	Domestic Cleaning	Comm. & Support Services
	Pharmacy Consultant (high)	Allied Health Services	Continence (pads)	Consumables	Social Support	Comm. & Support Services
	Physiotherapist (high)	Allied Health Services	Transport	Individual Transport		
	Shopping (high)	Comm. & Support Services	Nursing	Nursing Services		
	Domestic Cleaning (med)	Comm. & Support Services				
	Meals (med)	Comm. & Support Services				
	Showering (med)	Comm. & Support Services				
	Home Modifications (med)	Comm. & Support Services				

**Budget percentage (of HCPL3 + ITF + Dem sup)**	**$3,175.05**	**Budget %**	**$**	**Budget %**	**$**	**Budget %**
Comm. & Support Services	$ 1,468.75	46.26%	$ 1,284.00	40.44%	$ 1,075.50	33.87%
Nursing Services	$ 285.50	8.99%	$ 404.75	12.75%	$ -	0.00%
Allied Health Services	$ 976.00	30.74%	$ 122.00	3.84%	$ -	0.00%
Transport Individual Vehicle Costs	$ -	0.00%	$ 84.00	2.65%	$ -	0.00%
Transport Group Vehicle Costs	$ -	0.00%	$ -	0.00%	$ -	0.00%
Centre Bus Travel	$ -	0.00%	$ -	0.00%	$ -	0.00%
Brokered Services	$ -	0.00%	$ -	0.00%	$ -	0.00%
Consumables	$ -	0.00%	$ 319.70	10.07%	$ 176.00	5.54%
Fees	$ -	0.00%	$ 669.25	21.08%	$ 669.25	21.08%
TOTAL	$ 2,730.25	85.99%	$ 2,883.70	90.82%	$ 1,920.75	60.50%

**Proportional Allocation**			
Comm. & Support Services	53.80%	58%	86%
Nursing Services	10.46%	18%	0%
Allied Health Services	35.75%	6%	0%
Transport Individual Vehicle Costs	0.00%	4%	0%
Transport Group Vehicle Costs	0.00%	0%	0%
Centre Bus Travel	0.00%	0%	0%
Brokered Services	0.00%	0%	0%
Consumables	0.00%	14%	14%
CHECK	100.00%	100.00%	100.00%

**Unmet Needs**	**$**	**L2**	**L4**
Funding Allocation			
Comm. & Support Services			
Nursing Services			
Allied Health Services			
Transport Individual Vehicle Costs			
Transport Group Vehicle Costs			
Centre Bus Travel			
Brokered Services			
Consumables			
TOTAL	$ 2,730.25	$ -	
Budget deficit/surplus		-$ 2,730.25	-$ 2,730.25

ID	ACAT Recommendation (priority)	Service Category	Budget Allocation Discussion	Service Category	Resource Allocation Decision	Service Category
630860	Home Care Package L3 (high)	n/a	Fees	n/a	HCP l4 Alt Prov	Funding
	Home Modifications (high)	Home Modifications (high)	Existing services	n/a	Nursing (medications)	Nursing Services
	Gardening (high)	Gardening (high)	Gardening	Comm. & Support Services	Personal Alarm	Equipment
	Repairs (high)	Repairs (high)	Cleaning	Comm. & Support Services	Mowing	Comm & Support Services
	Social group (high)	Social group (high)	Shopping	Comm. & Support Services	Gardening	Comm & Support Services
	Social Support (med)	Social Support (med)	Social Activities	Comm. & Support Services		
	Transport (med)	Transport (med)	Transport	Comm. & Support Services		
	Residential Aged Care (low)	n/a	Eye Specialist	Allied Health Services		
			Social support	Comm. & Support Services		
			Personal Alarms	Equipment		
			Respite	Comm. & Support Services		
			Nursing (for injections)	Nursing Services		
			Mowing	Comm. & Support Services		
			Webster Packs	Allied Health Services		
			Physiotherapy	Allied Health Services		
			Podiatrist	Allied Health Services		
			Home Modifications	Allied Health Services		

**Budget percentage (of HCPL4)**	**$4,317.34**	**Budget %**	**$**	**Budget %**	**$**	**Budget %**
Comm. & Support Services	$ 691.75	16.02%	$ 1,688.00	39.10%	$ 216.75	5.02%
Nursing Services	$ -	0.00%	$ 1,423.13	32.96%	$ 1,423.13	32.96%
Allied Health Services	$ -	0.00%	$ 879.75	20.38%	$ -	0.00%
Transport Individual Vehicle Costs	$ 168.00	3.89%	$ 168.00	3.89%	$ -	0.00%
Transport Group Vehicle Costs	$ -	0.00%	$ -	0.00%	$ -	0.00%
Centre Bus Travel	$ -	0.00%	$ -	0.00%	$ -	0.00%
Brokered Services	$ -	0.00%	$ -	0.00%	$ -	0.00%
Consumables	$ -	0.00%	$ -	0.00%	$ -	0.00%
Fees	$ -	0.00%	$ 1,014.53	23.50%	$ 1,014.53	23.50%
TOTAL	$ 859.75	19.91%	$ 5,173.41	119.83%	$ 2,654.41	61.48%

**Proportional Allocation**			
Comm. & Support Services	80.46%	41%	13%
Nursing Services	0.00%	34%	87%
Allied Health Services	0.00%	21%	0%
Transport Individual Vehicle Costs	19.54%	4%	0%
Transport Group Vehicle Costs	0.00%	0%	0%
Centre Bus Travel	0.00%	0%	0%
Brokered Services	0.00%	0%	0%
Consumables	0.00%	0%	0%
CHECK	100.00%	100.00%	100.00%

**Unmet Needs**	$			**Equipment:**
Funding Allocation				Personal Alarm
Comm. & Support Services				
Nursing Services				
Allied Health Services				
Transport Individual Vehicle Costs				
Transport Group Vehicle Costs				
Centre Bus Travel				
Brokered Services				
Consumables				
TOTAL		$ -		
Budget deficit/surplus		$ -	$ -	

ID	ACAT Recommendation (priority)	Service Category	Budget Allocation Discussion	Service Category	Resource Allocation Decision	Service Category
795603	Home Care Package L2 (med)	n/a	Fees	n/a	In home respite 3 hours/fn	Comm. & Support Services
	Respite (low)	Comm. & Support Services	Upgrade package	n/a	Domestic Cleaning 1.5 hrs/wk	Comm. & Support Services
	Minor modifications (low)	Comm. & Support Services	Taxi vouchers	Consumables	Dementia consultant	Nursing Services
	Nursing (low)	Nursing Services	Dementia consultant	Nursing Services		
	Domestic Cleaning (low)	Comm. & Support Services	Existing services	Comm. & Support Services		
			Additional Services	Comm. & Support Services		
			Respite	Comm. & Support Services		
			Incontinence pads	Consumables		
			Laundry	Comm. & Support Services		
			Package for carer	n/a		
			Mobility equipment	Equipment		
**Budget percentage (of HCPL2)**	**$1,308.83**	**Budget %**	**$**	**Budget %**	**$**	**Budget %**
Comm. & Support Services	$ 843.25	64.43%	$ 1,204.00	91.99%	$ 771.00	58.91%
Nursing Services	$ 119.25	9.11%	$ 119.25	9.11%	$ 119.25	9.11%
Allied Health Services	$ -	0.00%	$ -	0.00%	$ -	0.00%
Transport Individual Vehicle Costs	$ -	0.00%	$ -	0.00%	$ -	0.00%
Transport Group Vehicle Costs	$ -	0.00%	$ -	0.00%	$ -	0.00%
Centre Bus Travel	$ -	0.00%	$ -	0.00%	$ -	0.00%
Brokered Services	$ -	0.00%	$ -	0.00%	$ -	0.00%
Consumables	$ -	0.00%	$ 253.70	19.38%	$ -	0.00%
Fees	$ -	0.00%	$ 307.58	23.50%	$ 307.58	23.50%
TOTAL	$ 962.50	73.54%	$ 1,884.53	143.99%	$ 1,197.83	91.52%
**Proportional Allocation**						
Comm. & Support Services	87.61%		76%		87%	
Nursing Services	12.39%		8%		13%	
Allied Health Services	0.00%		0%		0%	
Transport Individual Vehicle Costs	0.00%		0%		0%	
Transport Group Vehicle Costs	0.00%		0%		0%	
Centre Bus Travel	0.00%		0%		0%	
Brokered Services	0.00%		0%		0%	
Consumables	0.00%		16%		0%	
CHECK	100.00%	0.00%	100.00%	0.00%	100.00%	
**Unmet Needs**	**$**					
Funding Allocation						
Comm. & Support Services						
Nursing Services						
Allied Health Services						
Transport Individual Vehicle Costs						
Transport Group Vehicle Costs						
Centre Bus Travel						
Brokered Services						
Consumables						
TOTAL		$ -				
Budget deficit/surplus		$ -	$ -			

ID	ACAT Recommendation (priority)	Service Category	Budget Allocation Discussion	Service Category	Resource Allocation Decision	Service Category
830024	Home Care Package L2 (med)	n/a	Existing Services	n/a	Showers	Comm. & Support Services
	Personal Care (low)	Comm. & Support Services	Showers	Comm. & Support Services	Meals on wheels	Consumables
	Social Support (Low)	Comm. & Support Services	Social Support	Comm. & Support Services		
	Equipment (low)	Equipment	Physiotherapy	Allied Health Services		
	Meals (low)	Comm. & Support Services	Domestic Cleaning	Comm. & Support Services		
	Respite (med)	n/a	Continence pads	Consumables		
			Meals	Consumables		
			Fees and OOP costs	Fees		
			Respite	n/a		
			Carer consistency	n/a		

**Budget percentage (of HCPL1)**	**$743.99**	**Budget %**	**$**	**Budget %**	**$**	**Budget %**
Comm. & Support Services	$ 1,019.25	137.00%	$ 818.50	110.01%	$ 360.75	48.49%
Nursing Services	$ -	0.00%	$ -	0.00%	$ -	0.00%
Allied Health Services	$ -	0.00%	$ 488.00	65.59%	$ -	0.00%
Transport Individual Vehicle Costs	$ -	0.00%	$ -	0.00%	$ -	0.00%
Transport Group Vehicle Costs	$ -	0.00%	$ -	0.00%	$ -	0.00%
Centre Bus Travel	$ -	0.00%	$ -	0.00%	$ -	0.00%
Brokered Services	$ -	0.00%	$ -	0.00%	$ -	0.00%
Consumables	$ -	0.00%	$ 319.70	42.97%	$ 176.00	23.66%
Fees	$ -	0.00%	$ 174.84	23.50%	$ 174.84	23.50%
TOTAL	$ 1,019.25	137.00%	$ 1,801.04	242.08%	$ 711.59	95.65%

**Proportional Allocation**			
Comm. & Support Services	100.00%	50%	67%
Nursing Services	0.00%	0%	0%
Allied Health Services	0.00%	30%	0%
Transport Individual Vehicle Costs	0.00%	0%	0%
Transport Group Vehicle Costs	0.00%	0%	0%
Centre Bus Travel	0.00%	0%	0%
Brokered Services	0.00%	0%	0%
Consumables	0.00%	20%	33%
CHECK	100.00%	100.00%	100.00%

**Unmet Needs**	**$**	**L2**	**L1**
Funding Allocation		$ 1,308.83	$ 743.99
Comm. & Support Services			
Nursing Services			
Allied Health Services			
Transport Individual Vehicle Costs			
Transport Group Vehicle Costs			
Centre Bus Travel			
Brokered Services			
Consumables			
TOTAL	$ 1,019.25	$ -	
Budget deficit/surplus		$ 289.58	-$ 275.26

ID	ACAT Recommendation (priority)	Service Category	Budget Allocation Discussion	Service Category	Resource Allocation Decision	Service Category
830937	Home Care Package L2	n/a	Meals on wheels	Consumables	Mowing	Comm. & Support Services
	Nursing	Nursing Services	Shopping as a social activity	Comm. & Support Services	Social visits	Comm. & Support Services
	Social Support (high)	Comm. & Support Services	Dementia Consultant	Nursing Services	Shopping	Comm. & Support Services
	Occupational Therapist (high)	Allied Health Services	Respite care	n/a		
	Respite Care (high)	n/a	Transport	Transport Individual		
	Physiotherapy	Allied Health Services	Social support	Social Support		
	Meal Preparation	Comm. & Support Services	Equipment	Equipment		
	Transport (med)	Comm. & Support Services	Mowing	Comm. & Support Services		
	Home mods (med)	Comm. & Support Services	Incontinence aids	Consumables		
	Residential Aged Care (low)	n/a				

**Budget percentage (of HCPL2+dem)**	**$1,459.39**	**Budget %**	**$**	**Budget %**	**$**	**Budget %**
Comm. & Support Services	$ 851.00	58.31%	$ 778.75	53.36%	$ 602.25	41.27%
Nursing Services	$ 856.50	58.69%	$ 119.25	8.17%		0.00%
Allied Health Services	$ 610.00	41.80%	$ -	0.00%		0.00%
Transport Individual Vehicle Costs	$ 84.00	5.76%	$ 84.00	5.76%		0.00%
Transport Group Vehicle Costs	$ -	0.00%	$ -	0.00%		0.00%
Centre Bus Travel	$ -	0.00%	$ -	0.00%		0.00%
Brokered Services	$ -	0.00%	$ -	0.00%		0.00%
Consumables	$ -	0.00%	$ 319.70	21.91%		0.00%
Fees	$ -	0.00%	$ 307.58	21.08%	$ 307.58	21.08%
TOTAL	$ 2,401.50	164.56%	$ 1,609.28	110.27%	$ 909.83	62.34%

**Proportional Allocation**			
Comm. & Support Services	35.44%	60%	100%
Nursing Services	35.67%	9%	0%
Allied Health Services	25.40%	0%	0%
Transport Individual Vehicle Costs	3.50%	6%	0%
Transport Group Vehicle Costs	0.00%	0%	0%
Centre Bus Travel	0.00%	0%	0%
Brokered Services	0.00%	0%	0%
Consumables	0.00%	25%	0%
CHECK	100.00%	100%	100%

**Unmet Needs**	**$**	**L2**	**L2**	**Equipment**
Funding Allocation		$ 1,308.83	$ 1,308.83	
Comm. & Support Services	$ 851.00			
Nursing Services	$ 856.50			
Allied Health Services	$ 610.00			
Transport Individual Vehicle Costs	$ 84.00			
Transport Group Vehicle Costs				
Centre Bus Travel				
Brokered Services				
Consumables				
TOTAL	$ 2,401.50	$ -		
Budget deficit/surplus		-$ 1,092.67	-$ 1,092.67	

ID	ACAT Recommendation (priority)	Service Category	Budget Allocation Discussion	Service Category	Resource Allocation Decision	Service Category
879341	Social Support (high)	Comm. & Support Services	Fees	Fees	Showers 3 x 30min/week	Comm. & Support Services
	Podiatry (high)	Allied Health Services	Existing Services	sum	Cleaning 1.5hr/fn	Comm. & Support Services
	Continence Advisory Service (high)	Nursing Services	Showers	Comm. & Support Services	Day Respite (alt program)	Alt funding
	Occupational therapy (high)	Allied Health Services	Domestic Cleaning	Comm. & Support Services		
	Psychologist (high)	Allied Health Services	Shopping	Comm. & Support Services		
	Home Care Package L3 (med)	Funds	Compare Providers	n/a		
	Personal Care (med)	Comm. & Support Services	Gardening	Comm. & Support Services		
	Home Maintenance (med)	Comm. & Support Services	Transport	Comm. & Support Services		
	Domestic Cleaning (med)	Comm. & Support Services	Physiotherapist	Allied Health Services		
	Meals (low)	Comm. & Support Services	Medication Management	Allied Health Services		
	Transport (low)	Comm. & Support Services	Home Modifications	Comm. & Support Services		
	Respite (low)	n/a	Podiatry	Allied Health Services		
			Key Safe	Equipment		

**Budget percentage (of HCPL1)**	**$743.99**	**Budget %**	**$**	**Budget %**	**$**	**Budget %**
Comm. & Support Services	$ 900.00	120.97%	$ 1,211.75	162.87%	$ 561.50	75.47%
Nursing Services	$ 95.75	12.87%	$ -	0.00%		0.00%
Allied Health Services	$ 541.75	72.82%	$ 659.25	88.61%		0.00%
Transport Individual Vehicle Costs	$ 84.00	11.29%		0.00%		0.00%
Transport Group Vehicle Costs	$ -	0.00%		0.00%		0.00%
Centre Bus Travel	$ -	0.00%		0.00%		0.00%
Brokered Services	$ -	0.00%		0.00%		0.00%
Consumables	$ -	0.00%		0.00%		0.00%
Fees	$ -	0.00%	$ 174.84	23.50%	$ 174.84	23.50%
TOTAL	$ 1,621.50	217.95%	$ 2,045.84	274.98%	$ 736.34	0.00%

**Proportional Allocation**			
Comm. & Support Services	55.50%	65%	100%
Nursing Services	5.91%	0%	0%
Allied Health Services	33.41%	35%	0%
Transport Individual Vehicle Costs	5.18%	0%	0%
Transport Group Vehicle Costs	0.00%	0%	0%
Centre Bus Travel	0.00%	0%	0%
Brokered Services	0.00%	0%	0%
Consumables	0.00%	0%	0%
CHECK	100.00%	100%	100%

**Unmet Needs**	**$**	**L3**	**L1**	**Equipment**	
Funding Allocation		$ 2,847.91	$ 743.99	Key Safe	$100 supply and install
Comm. & Support Services	$ 900.00				
Nursing Services	$ 95.75				
Allied Health Services	$ 541.75				
Transport Individual Vehicle Costs	$ 84.00				
Transport Group Vehicle Costs					
Centre Bus Travel					
Brokered Services					
Consumables					
TOTAL	$ 1,621.50	$ -			
Budget deficit/surplus		$ 1,226.41	-$ 877.51		

ID	ACAT Recommendation (priority)	Service Category	Budget Allocation Discussion	Service Category	Resource Allocation Decision	Service Category
916234	Personal Care (high)	Comm. & Support Services	Compare Providers	n/a	Pkg with Alt Prov	
	Nursing (med)	Nursing Services	Existing Services	Comm. & Support Services	Services yet to commence	
	Respite (med)	n/a	Budget and Fees	Fees		
	Home Modifications (med)	Comm. & Support Services	Meals	Consumables		
	Occupational Therapy (med)	Allied Health Services	Self-management	Fees		
	Residential Aged Care (low)	n/a	Social Support	Comm. & Support Services		
	Shopping (low)	Comm. & Support Services	Mowing	Comm. & Support Services		
	Domestic Cleaning (low)	Comm. & Support Services				
	Meals (low)	Comm. & Support Services				
	Social Support (low)	Comm. & Support Services				

Budget percentage (of HCPL4 + dem)	$4,813.74	Budget %	$	Budget %	$	Budget %
Comm. & Support Services	$ 1,380.50	28.68%	$ 1,019.25	21.17%	$ -	0.00%
Nursing Services	$ 856.50	17.79%	$ -	0.00%	$ -	0.00%
Allied Health Services	$ 122.00	2.53%	$ -	0.00%	$ -	0.00%
Transport Individual Vehicle Costs	$ -	0.00%	$ -	0.00%	$ -	0.00%
Transport Group Vehicle Costs	$ -	0.00%	$ -	0.00%	$ -	0.00%
Centre Bus Travel	$ -	0.00%	$ -	0.00%	$ -	0.00%
Brokered Services	$ -	0.00%	$ -	0.00%	$ -	0.00%
Consumables	$ -	0.00%	$ 176.00	3.66%	$ -	0.00%
Fees	$ -	0.00%	$ 1,014.57	21.08%	$ 1,014.57	21.08%
TOTAL	$ 2,359.00	49.01%	$ 2,209.82	45.91%	$ 1,014.57	21.08%

Proportional Allocation			
Comm. & Support Services	58.52%	85%	0%
Nursing Services	36.31%	0%	0%
Allied Health Services	5.17%	0%	0%
Transport Individual Vehicle Costs	0.00%	0%	0%
Transport Group Vehicle Costs	0.00%	0%	0%
Centre Bus Travel	0.00%	0%	0%
Brokered Services	0.00%	0%	0%
Consumables	0.00%	15%	0%
CHECK	100.00%	100%	0%

Unmet Needs	$		
Funding Allocation			
Comm. & Support Services			
Nursing Services			
Allied Health Services			
Transport Individual Vehicle Costs			
Transport Group Vehicle Costs			
Centre Bus Travel			
Brokered Services			
Consumables			
TOTAL	$ 2,359.00	$ -	
Budget deficit/surplus		-$ 2,359.00	-$ 2,359.00

ID	ACAT Recommendation (priority)	Service Category	Budget Allocation Discussion	Service Category	Resource Allocation Decision	Service Category
959965	Home Care Package L2 (med)	funds	Existing services	sum	Showering	Comm. & Support Services
	Home Modification (high)	Comm. & Support Services	Showers	Comm. & Support Services	Cleaning (brokered)	Brokered Services
	Transport (high)	Transport	Domestic cleaning (f/n)	Comm. & Support Services	Landscaping (brokered)	Brokered Services
	Occupational Therapy (high)	Allied Health Care At Home	Missing Services	sum	Case Management	Fees
	Nursing (high)	Allied Health Care At Home	Gardening	Comm. & Support Services		
	Continence Advisory Service (high)	Nursing Services	Continence Aids	consumables		
	Domestic Cleaning (high)	Comm. & Support Services	More showers	Comm. & Support Services		
	Personal Care (high)	Comm. & Support Services	Shopping	Comm. & Support Services		
			Transport	Transport Individual		
			Podiatry	Allied Health Care At Home		
			Fees	fees		

Budget percentage (of HCPL2)	$1,308.83	Budget %	$	Budget %	$	Budget %
Comm. & Support Services	$ 810.25	61.91%	$ 1,380.00	105.44%	$ 360.75	27.56%
Nursing Services	$ 404.75	30.92%	$ -	0.00%	$ -	0.00%
Allied Health Services	$ 122.00	9.32%	$ 72.75	5.56%	$ -	0.00%
Transport Individual Vehicle Costs	$ 84.00	6.42%	$ 84.00	6.42%	$ -	0.00%
Transport Group Vehicle Costs	$ -	0.00%	$ -	0.00%	$ -	0.00%
Centre Bus Travel	$ -	0.00%	$ -	0.00%	$ -	0.00%
Brokered Services	$ -	0.00%	$ -	0.00%	$ 478.50	36.56%
Consumables	$ -	0.00%	$ -	0.00%	$ -	0.00%
Fees	$ -	0.00%	$ 307.58	23.50%	$ 307.58	23.50%
TOTAL	$ 1,421.00	108.57%	$ 1,844.33	140.91%	$ 1,146.83	87.62%

**Proportional Allocation**			
Comm. & Support Services	57.02%	90%	43%
Nursing Services	28.48%	0%	0%
Allied Health Services	8.59%	5%	0%
Transport Individual Vehicle Costs	5.91%	5%	0%
Transport Group Vehicle Costs	0.00%	0%	0%
Centre Bus Travel	0.00%	0%	0%
Brokered Services	0.00%	0%	57%
Consumables	0.00%	0%	0%
CHECK	100.00%	100.00%	100.00%

**Unmet Needs**	$		
Funding Allocation			
Comm. & Support Services			
Nursing Services			
Allied Health Services			
Transport Individual Vehicle Costs			
Transport Group Vehicle Costs			
Centre Bus Travel			
Brokered Services			
Consumables			
TOTAL	$ 1,421.00	$ -	
Budget deficit/surplus		-$ 1,421.00	-$ 1,421.00
